# A rare case of small-cell neuroendocrine tumour of the lung metastasising to the urinary bladder

**DOI:** 10.4102/sajr.v27i1.2556

**Published:** 2023-04-26

**Authors:** Humphrey Mapuranga, Siseko Silolo, Abraham C. van Wyk, Sucari S.C. Vlok

**Affiliations:** 1 Department of Radiodiagnosis, Faculty of Medicine and Health Sciences, Stellenbosch University, Cape Town, South Africa; 2 Department of Urology, Faculty of Medicine and Health Sciences, Stellenbosch University, Cape Town, South Africa; 3 Division of Anatomical Pathology, Faculty of Medicine and Health Sciences, National Health Laboratory Service, Stellenbosch University, Cape Town, South Africa

**Keywords:** small-cell neuroendocrine carcinoma, metastases, cystoscopy, immunohistochemistry, biopsy

## Abstract

A 77-year-old woman with suspected lung carcinoma had multiple bladder masses and lymphadenopathy outside the normal urinary bladder drainage area. Fine needle aspiration and immunocytochemistry of the cervical lymph node complex and transurethral biopsy of the bladder masses confirmed metastatic small-cell neuroendocrine carcinoma.

**Contribution:**

Clinical correlation, imaging findings, tumour markers and immunohistochemistry are necessary for metastatic bladder tumour work-up.

## Introduction

Urinary bladder metastases are rare, accounting for 4.5% of all bladder neoplasms. Secondary bladder neoplasms can arise from direct extension from another pelvic malignancy or from distant organs as part of a more extensive disease.^1^ Primary malignancies with distant spread to the urinary bladder include the stomach, skin, lung and breast.^2^ Bladder metastases from a proven primary lung neoplasm are extremely rare with a paucity of literature on this specific entity.

Owing to the uncommon occurrence of distant bladder metastases from a non-contiguous primary source and the morphologic diversity of urothelial carcinoma of the bladder, accurate diagnosis can be challenging.

## Case presentation

A 77-year-old woman known with hypertension, chronic obstructive pulmonary disease and a strong smoking history of 20 pack years, presented with a 4-month history of dysphagia, odynophagia and dysphonia. She reported chronic loss of appetite and tolerated only fluid feeds with hesitancy for solids. She had significant weight loss of approximately 20 kg over a 3-month period. At the time of presentation, she had no haematuria, flank pain, dysuria or any accompanying genitourinary symptoms. She had a previous hysterectomy and a surgical procedure for perforated peptic ulcer disease.

On physical examination, she was frail and cachectic with poor performance status: Eastern Cooperative Oncology Group grade 4 (ECOG-4). On clinical examination, a 3 cm firm, fixed and tender right cervical lymph node complex was palpated. Chest and breast examinations were normal. Review of other systems was unremarkable.

**FIGURE 1:**
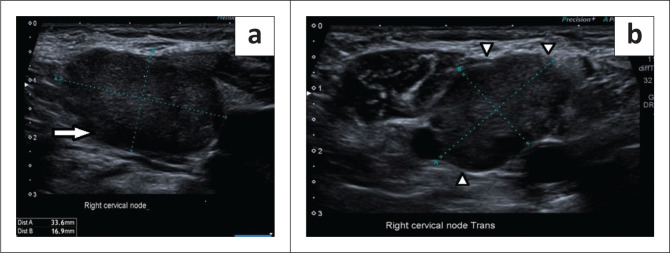
Right cervical pathological lymphadenopathy (a) Longitudinal and (b) transverse ultrasound images demonstrated loss of the fatty hila (solid white arrow), lobulated contours (solid white arrow heads) and increased short axis diameter of 1.69 cm.

**FIGURE 2:**
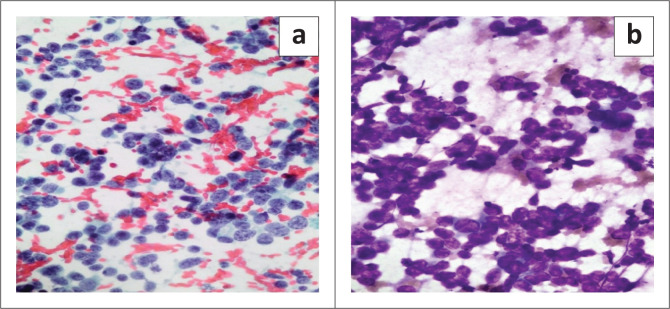
Fine needle aspiration biopsy of the cervical lymph node showed poorly cohesive malignant cells with a high nuclear-cytoplasmic ratio, scant cytoplasm, ‘salt-and-pepper’ chromatin and inconspicuous nucleoli similar to the cells seen in the urinary bladder. Papanicolaou stain, original magnification 400x (a) and Diff Quik stain, original magnification 400x (b).

Gastroscopy demonstrated extrinsic oesophageal compression at 30 cm. Right neck ultrasound revealed a cluster of large, lobulated, hypoechoic lymph nodes with loss of fatty hila (Figure 1a & b). A lymph node fine needle aspiration biopsy proved metastatic carcinoma with morphological features suggestive of small-cell carcinoma (Figure 2a & b). Synaptophysin, chromogranin A and CD56 immunocytochemical stains were positive on the cell block, confirming neuroendocrine differentiation.

**FIGURE 3:**
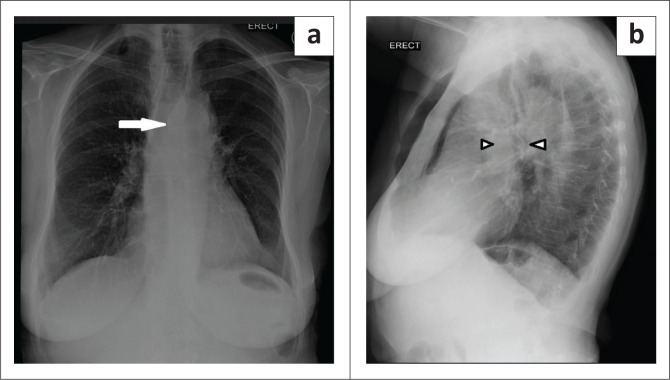
(a) PA CXR: Left hilar soft tissue mass with attenuation of the left main-stem bronchus and right tracheal shift (white arrow). Lateral CXR (b) shows hilar and mediastinal adenopathy (white arrow heads).

**FIGURE 4:**
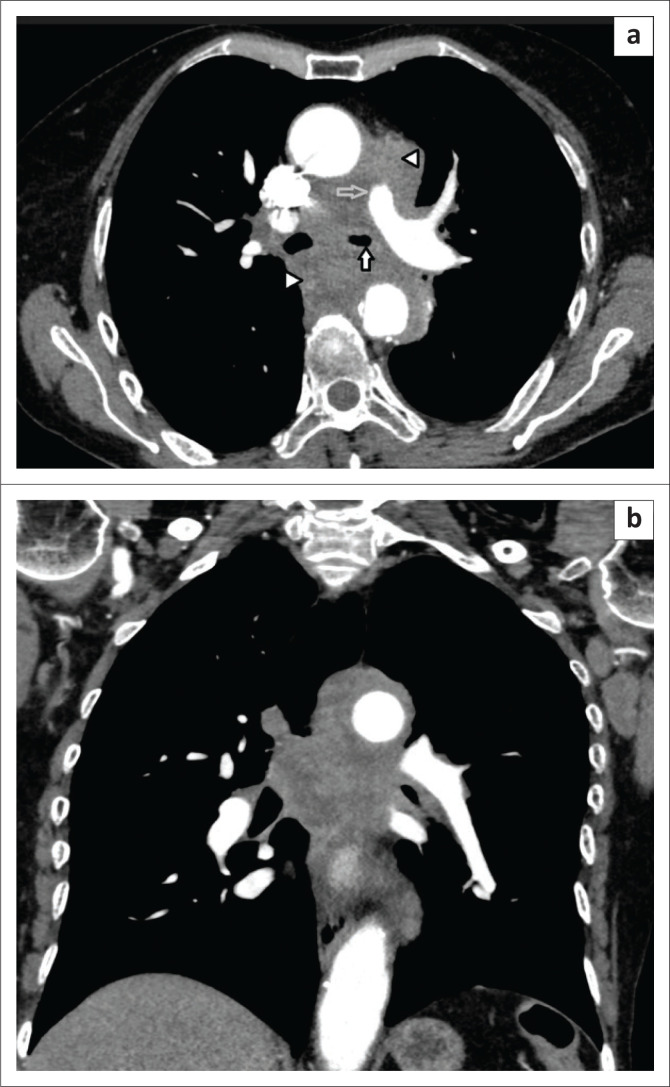
(a) CT chest axial: Confirms extensive hilar and mediastinal mass-nodal complex with necrosis (white arrow heads). The mass encases and narrows the left mainstem bronchus (solid white arrow), main pulmonary trunk (grey arrow), and (b) coronal reconstruction shows trans-spatial extent of the mass.

**FIGURE 5:**
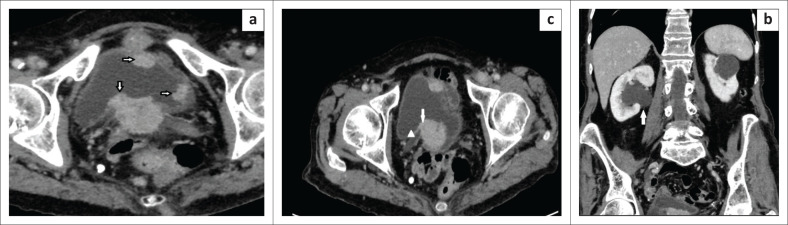
Abdomino-pelvic CT (a) multiple urinary bladder enhancing exophytic mural mass lesions (white arrows) (b) lesion (white arrow) located in the posterior bladder wall infiltrates the right vesico-ureteric junction (white arrow head), causing upstream obstructive uropathy (c) dilatation of the right renal pelvis (white arrow) secondary to tumour infiltrating the right vesico-ureteric junction.

A posteroanterior chest radiograph performed at presentation revealed a left hilar mass with attenuation of the left main-stem bronchus and accompanying contralateral mediastinal shift (Figure 3). Post-contrast staging chest CT (Figure 4) showed an infiltrative, central, trans-spatial mediastinal mass encasing the left main-stem bronchus, left and right main pulmonary arteries and descending aorta. Abdomino-pelvic CT revealed multiple enhancing soft tissue mural lesions in the urinary bladder – consistent with metastases – the largest was located at the bladder base, with a similar lesion located anteriorly – in the space of Retzius – (Figure 5a), and complicated by right-sided hydro-uretero-nephrosis because of infiltration of the right vesico-ureteric junction (VUJ) by the largest deposit (Figure 5b & c). There was no abdominopelvic nodal or solid organ metastatic disease. No enhancing nodules were present in the right renal pelvis. The axial and appendicular skeletal elements were clear of metastases.

**FIGURE 6:**
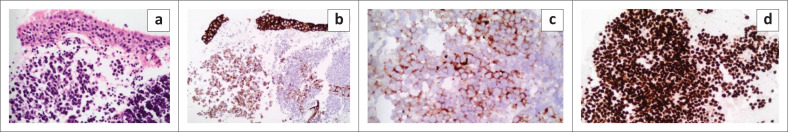
Histological and immunohistochemical findings in the bladder biopsy. (a) Malignant cells are present in the lamina propria (left lower quadrant) with overlying normal urothelium and a lymphocytic aggregate to the right (haematoxylin and eosin, original magnification 200x). The tumour cells and urothelium express cytokeratins (MNF116) (b). Synaptophysin is expressed in the cytoplasm of the tumour cells (c) and thyroid transcription factor 1 is strongly expressed in the tumour cell nuclei (d).

At this point, the patient was discussed at a multidisciplinary meeting and the diagnosis of central bronchus carcinoma (small-cell) with possible metastases to the urinary bladder was made. The cancer was staged radiologically as T4N3M1c. The patient was referred to the urology team for further workup of the bladder lesions and management of the right-sided hydro-uretero-nephrosis. Cystoscopy revealed several solid broad-based masses, of which the mass along the right posterior bladder wall was biopsied. Histology of the biopsy specimen revealed a small round blue cell tumour in the lamina propria with normal overlying urothelium and accompanying lymphoid aggregates. Immunohistochemical stains for broad-spectrum cytokeratin (MNF116), synaptophysin, chromogranin A and TTF-1 were all positive, confirming the diagnosis of metastatic small-cell neuroendocrine carcinoma (Figure 6a, b, c & d).

Correlating the clinical, radiological and pathological findings, a final diagnosis of small-cell neuroendocrine carcinoma of the lung with metastases to the urinary bladder was made.

## Management and outcome

Given the patient’s extensive disease burden, metastatic spread and poor performance status (ECOG-4), the decision was taken by the family in conjunction with the oncology team to place the patient on palliative care. The patient was discharged and hospice care was arranged. The patient unfortunately succumbed 6 months after the initial presentation.

## Discussion

Neuroendocrine tumours consist of a large heterogeneous group of malignancies derived from embryonic neural crest tissue found in various organ systems. Four major types of lung neuroendocrine tumours are described in the 2004 World Health Organization classification: typical carcinoid, atypical carcinoid, large cell neuroendocrine carcinoma and small-cell lung cancer.

Neuroendocrine tumours make up 25% of primary lung carcinomas with the most common of these being small-cell carcinoma. More than 95% of small-cell carcinomas arise from the lung, implying that a primary lung lesion should be sought for if a lesion, which is histologically confirmed as small-cell carcinoma is found within any other organ system.^3^

Small-cell neuroendocrine carcinomas (SNECs) of the lung are high-grade, poorly differentiated malignancies, known to be highly aggressive. They tend to arise near the bronchial region in the majority of cases. These tumours carry a poor prognosis and once diagnosed, local or distant metastases from the primary site are usually present. The mean age at presentation is 65 years, and a strong association with heavy cigarette smoking is well known.^4^ Although neuroendocrine tumours of the urinary bladder are much less common compared with lung neuroendocrine tumours, it is essential to consider this differential diagnosis in uropathology as this is central in guiding drug development and patient management. Bladder neuroendocrine tumours are less common than the other conventional histological genitourinary variants, namely urothelial carcinoma, adenocarcinoma and squamous cell carcinoma, constituting only 0.7% – 1% of malignant bladder cancers.^5,6^ They can present with haematuria and pelvic pain.

Secondary neoplasms to the urinary bladder have been known to be extremely rare. In one series of 11 cases with bladder metastases, it was noticed that the most common primary tumour was the breast. Interestingly, in the same series, none of the patients had lung carcinoma as a primary site. Of the patients in this series, 51% demised within 1 year after the diagnosis of bladder metastases.^7^

The radiological features of secondary bladder tumours have not been extensively described in the relevant literature. The imaging features of secondary bladder tumours closely resemble primary bladder cancers. Differentiation on imaging alone is nearly impossible with tissue sampling, immunohistochemistry and clinical history often being necessary.^8^ The diagnosis of SNEC is confirmed when chromogranin, synaptophysin or CD56 are positive. In the presented case, the lymph node biopsy was also positive for all these markers.

It is interesting to note that small-cell carcinoma of the urinary bladder is histologically indistinguishable from small-cell carcinoma of the lung. This has led to the proposal that a shared pathogenesis in these entities exists. A research study, however, concluded that small-cell cancers of the bladder and lung share a convergent but distinct pathogenesis and in fact the former actually arises from a cell of origin shared with urothelial bladder cancer.^9^

Patients with primary small-cell carcinoma of the urinary bladder require aggressive combination therapy, such as combined chemotherapy and radical cystectomy or chemotherapy and radiation therapy, to achieve cure. Those with secondary small-cell bladder cancer are usually treated with palliative chemotherapy. A cisplatin-based chemotherapy regimen is the preferred initial therapy for patients with metastatic urothelial cancer of the bladder and urinary tract, but cisplatin-related toxicity is a major drawback to this regimen, especially in patients with diminished renal function and poor performance status.^10^

## Conclusion

While metastatic bladder cancers are rare and typically arise from contiguous sites, non-contiguous primary cancers can metastasise to the urinary bladder, although very few have been described in the literature. In particular, small-cell lung neuroendocrine cancer is extremely rare. In the workup of patients with bladder masses in the presence of a known primary, immunohistochemistry, histology and cross-sectional imaging are important in differentiating metastases to the bladder from a synchronous carcinoma and for assessing tumour genotype.
